# Supplementary material for: IL-1 Superfamily Across 400+ Species: Therapeutic Targets and Disease Implications
Source: Biology (Basel). 2025 May 17;14(5):561. doi: 10.3390/biology14050561 (PMC12108812; doi:10.3390/biology14050561)
Supplement: Supplementary file 1 [file biology-14-00561-s001.zip › Figure S1.pdf]

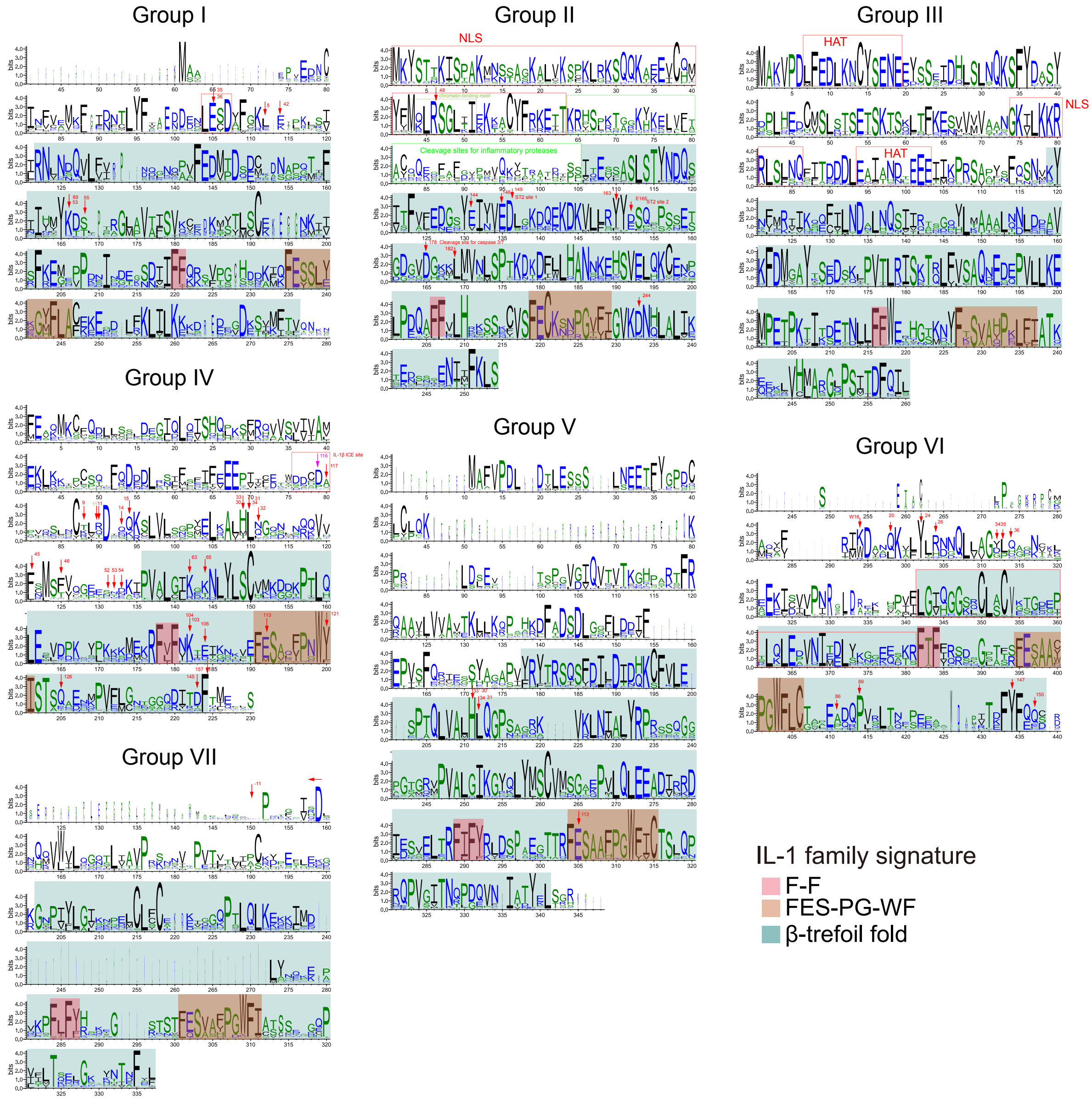
Group I
Group II
Group III
Group IV
Group V
Group VI
Group VII
NLS
HAT
chromatin-binding motif
Cleavage sites for inflammatory proteases
ST2 site 1
ST2 site 2
Cleavage site for caspase 3/7
IL-1β ICE site
IL-1 family signature
F-F
FES-PG-WF
β-trefoil fold
