# Supplementary figures and images for: IL-1 Superfamily Across 400+ Species: Therapeutic Targets and Disease Implications
Source: Biology (Basel). 2025 May 17;14(5):561. doi: 10.3390/biology14050561 (PMC12108812; doi:10.3390/biology14050561)

## Group I

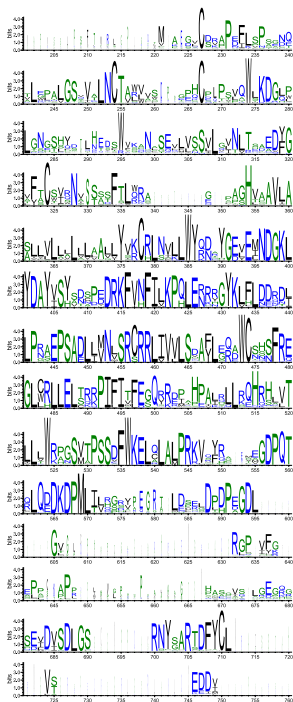

## Group II

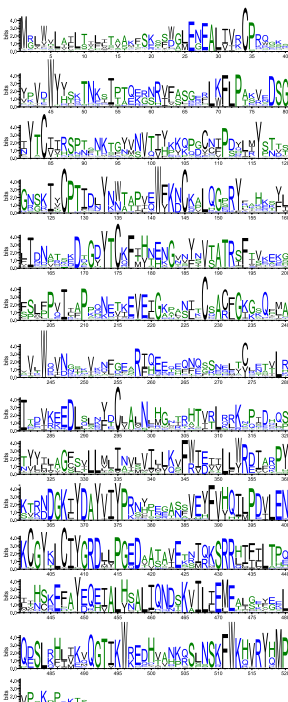

## Group III

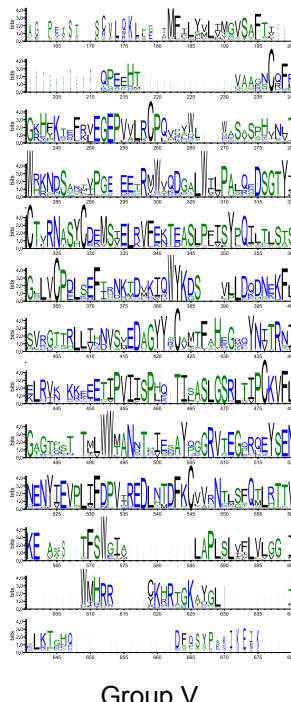

## Group IV

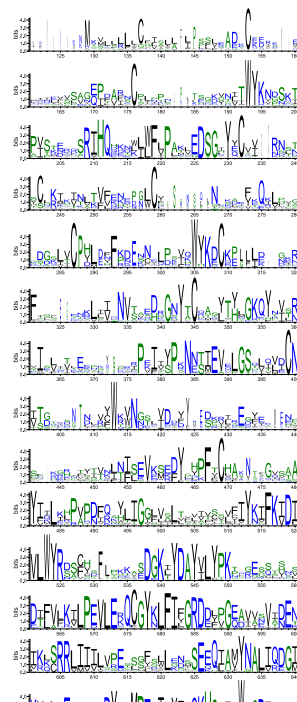

## Group VII

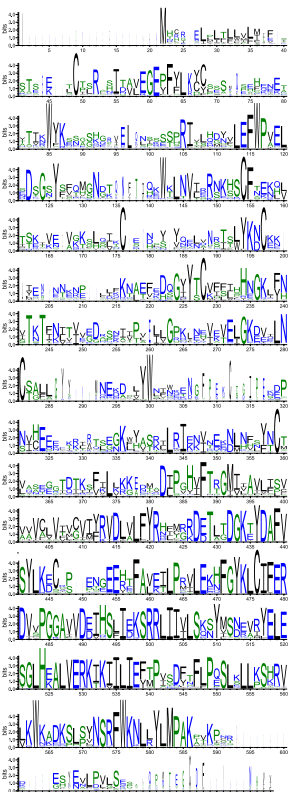

## Group VI

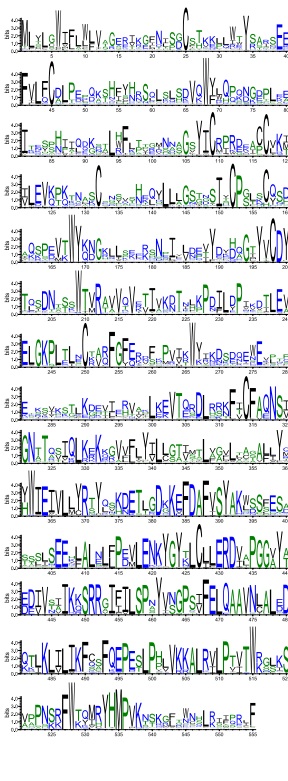

## Group V

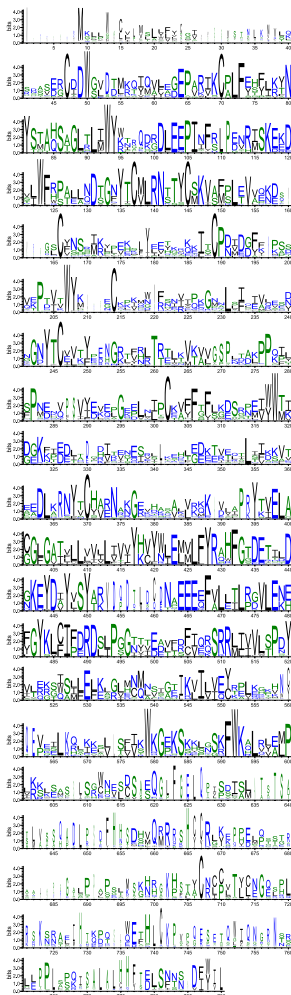

Supplement: Supplementary file 1 [file biology-14-00561-s001.zip › Figure S2.pdf]
